# *PECAM1*, *COL4A2*, *PHACTR1*, and *LMOD1* Gene Polymorphisms in Patients with Unstable Angina

**DOI:** 10.3390/jcm11020373

**Published:** 2022-01-13

**Authors:** Krzysztof Kosiński, Damian Malinowski, Krzysztof Safranow, Violetta Dziedziejko, Andrzej Pawlik

**Affiliations:** 1 Department of Cardiology, Hospital in Szczecin, Arkonska 4, 71-455 Szczecin, Poland; kriskos70@gmail.com; 2 Department of Experimental and Clinical Pharmacology, Pomeranian Medical University, 70-111 Szczecin, Poland; damian.malinowski@pum.edu.pl; 3 Department of Biochemistry and Medical Chemistry, Pomeranian Medical University, 70-111 Szczecin, Poland; chrissaf@mp.pl (K.S.); viola@pum.edu.pl (V.D.); 4 Department of Physiology, Pomeranian Medical University in Szczecin, Powstańców Wlkp. 72, 70-111 Szczecin, Poland

**Keywords:** coronary artery disease, unstable angina, polymorphism, genetic

## Abstract

Coronary artery disease (CAD) is a syndrome resulting from myocardial ischaemia of heterogeneous pathomechanism. Environmental and genetic factors contribute to its development. Atherosclerotic plaques that significantly narrow the lumen of coronary arteries cause symptoms of myocardial ischaemia. Acute coronary incidents are most often associated with plaque rupture or erosion accompanied by local activation of the coagulation system with thrombus formation. Plaque formation and stability are influenced by endothelial function and vascular smooth muscle cell function. In this study, we investigated the association between polymorphisms in genes affecting endothelial and vascular smooth muscle cell (VSMC) function and the occurrence of unstable angina pectoris. The aim of this study was to evaluate the association between the *PECAM1* (rs1867624), *COL4A2* (rs4773144), *PHACTR1* (rs9349379) and *LMOD1* (rs2820315) gene polymorphisms and the risk of unstable angina. The study included 232 patients with unstable angina diagnosed on the basis of clinical symptoms and coronary angiography and 144 healthy subjects with no significant coronary lumen stenosis at coronary angiography. There were no statistically significant differences in the distribution of *COL4A2 rs4773144* and *PECAM1 rs1867624 gene polymorphisms between patients with unstable angina and control subjects. In patients with unstable angina, there was an increased frequency of PHACTR1 rs9349379 G allele carriers (GG and AG genotypes)* (GG+AG vs. AA, OR 1.71; 95% CI 1.10–2.66, *p* = 0.017) and carriers of the *LMOD1* rs2820315 T allele (TT and CT genotypes) (TT+CT vs. CC, OR 1.65; 95% CI 1.09–2.51, *p* = 0.019) compared to the control group. The association between these alleles and unstable angina was confirmed by multivariate logistic regression analysis, in which the number of G (*PHACTR1* rs9349379) and T (*LMOD1* rs2820315) alleles was an independent risk factor for unstable angina. The results suggest an association between *PHACTR1* rs9349379 and *LMOD1* rs2820315 polymorphisms and the risk of unstable angina.

## 1. Introduction

Coronary artery disease (CAD) is a syndrome resulting from myocardial ischaemia of heterogeneous pathomechanism. In most cases, it is caused by atherosclerosis of the coronary arteries. Environmental and genetic factors contribute to its development. Factors such as smoking, hyperlipidaemia and disorders of lipid and carbohydrate metabolism are so-called modifiable risk factors. Genetic factors are still poorly understood, but recent years have brought great progress in this area. In particular, it concerns the identification of so-called single nucleotide polymorphisms (SNPs), which may be associated with the risk of CAD. Currently, more than 60 polymorphisms have been identified that may increase the risk of developing CAD [1].

CAD is a reflection of the systemic atherosclerotic process, which is a specific type of inflammation taking place within the inner membrane of the arterial walls. Initial atherosclerotic lesions consist mainly of macrophages transforming into foam cells and few lymphocytes [2]. With favourable environmental and genetic conditions, these primary lesions transform into more advanced and irreversible atherosclerotic plaques. In the process of the formation of mature atherosclerotic lesions, the following phenomena occur: migration of smooth muscle cells from the vascular membrane with their phenotypic reprogramming and proliferation, accumulation of extracellular matrix proteins, neovasculogenesis, apoptosis, activation of the coagulation system and calcification [3].

The inflammatory response is initiated and sustained by various pathological factors, among which oxidized LDL cholesterol particles (oxLDL) and heat shock proteins (HSP) seem to play a leading role. In response to the damaging factor, monocytes transmigrate from circulating blood to the inner membrane and are transformed into macrophages and then lipid-accumulating foam cells [4]. Endothelial dysfunction, which can be an initiating factor, as well as vascular smooth muscle cells, play a significant role in monocyte recruitment and platelet activation. Under the influence of antigen-presenting cells and the cytokines (such as IL-1 and TNF-α) and chemokines produced, the inflammatory process is further propagated through the involvement of specific immune mechanisms [5].

Atherosclerotic plaques that significantly narrow the lumen of coronary arteries cause symptoms of myocardial ischaemia. Acute coronary incidents are most often associated with plaque rupture or erosion accompanied by local activation of the coagulation system with thrombus formation [2]. They can critically restrict or completely prevent blood flow in the artery and consequently cause necrosis of a specific area of the myocardium. Atherosclerotic plaques prone to rupture, characterized by a large necrotic nucleus and a thin fibrous cap, are crucial in the etiopathogenesis of acute coronary syndromes [6]. Plaque formation and stability are influenced by endothelial function and vascular smooth muscle cell function. In this study, we investigated the association between polymorphisms in genes affecting endothelial and vascular smooth muscle cell (VSMC) function and the occurrence of unstable angina pectoris.

The phosphatase and actin regulator 1 (*PHACTR1*) gene is located on chromosomal locus 6p24. The rs9349379 polymorphism located within the intron of *PHACTR1* gene has been shown to affect its expression in vascular tissue [7]. *PHACTR1* regulates transcription factors responsible for extracellular signal transduction and activation of pathways that control differentiation, morphogenesis, proliferation, apoptosis of vascular endothelial cells and, consequently, atherogenesis and plaque stability [8,9,10]. The product of the *PHACTR1* gene is a protein phosphatase 1 (PP1—protein phosphatase 1) and actin binding protein. PP1 influences endothelial function and production of NO [11]. Previous studies have shown an association between the *PHACTR1* gene rs9349379 polymorphism and the severity of atherosclerotic lesions in the coronary arteries [12,13,14,15,16]. 

*LMOD1* (leiomodin 1) belongs to the actin filament neucleator family and is found in smooth muscle cells, including vascular smooth muscle cells [17]. The gene encoding this protein is located at locus 1q32. The rs2820315 polymorphism was shown to affect *LMOD1* gene expression in coronary artery smooth muscle cells [18]. *LMOD1* appears to play a key role in maintaining the phenotype of differentiated vascular smooth muscle cells and sustaining their contractile function [19]. Vascular muscle cells that have undergone phenotypic transformation produce excess of extracellular matrix, undergo apoptosis, accumulate lipids, secrete growth factors and proinflammatory cytokines, and contribute to monocyte recruitment [20]. Decreased expression of this gene leads to increased proliferation and migration as well as loss of contractile elements in smooth muscle cells of the coronary arteries and to increased atherosclerosis and plaque instability.

*PECAM1* (CD31) encodes platelet-endothelial cell adhesion factor 1, a trans-membrane glycoprotein belonging to the immunoglobulin family found on the surface of platelets, monocytes and neutrophils [21]. The *PECAM1* gene is located in the 17q23 region. *PECAM1* molecules concentrate on the lateral surfaces of adjacent cell membranes in the endothelium. Mutual binding of *PECAM1* molecules to each other through so-called transhomophilic connections leads to the formation of a specific protective barrier of the endothelium [22]. Down regulation of *PECAM1* expression leads to a violation of the integrity of the endothelial barrier and an increase in its permeability, which consequently lead to the activation of inflammatory processes in the arterial inner membrane [23]. The protective function of *PECAM1* is probably impaired at sites of active inflammation, i.e., in areas of arteries involved in the atherosclerotic process. The rs1867624 polymorphism has been shown to affect the expression of the PECAM gene and thus the formation of atherosclerotic lesions in the vessels and the development of coronary artery disease [24].

The COL4A1/*COL4A2* gene cluster, a member of the type IV collagen family, is located in region 13q34 and includes exons 52 and 48. This type of collagen is the main building block of the basement membrane, which connects the epithelial cell layer and vascular endothelium through integrins and other surface molecules [25]. SNP rs4773144 is located within intron 3 of the *COL4A2* gene. This polymorphism affects *COL4A2* gene expression and thus the development of atherosclerotic lesions [26]. Decreased *COL4A2* gene expression results in altered vascular smooth muscle cell phenotype, compromised endothelial barrier integrity, and apoptosis of vascular smooth muscle and endothelial cells [27]. Thus, type IV collagen deficiency promotes atherogenesis by altering the properties of vascular smooth muscle cells. In turn, weakening of the endothelial basement membrane with reduced collagen IV content facilitates monocyte recruitment from the circulation by increasing VCAM-1 expression Previous studies suggested an association between these polymorphisms and ischemic heart disease, but the results are inconsistent.

The aim of this study was to evaluate the association between the *PECAM1* (rs1867624), *COL4A2* (rs4773144), *PHACTR1* (rs9349379) and *LMOD1* (rs2820315) gene polymorphisms and the risk of unstable angina.

## 2. Material and Methods

The study included 232 patients with unstable angina treated in the Department of Cardiology in years 2017–2018. The diagnosis of unstable angina was made on the basis of typical clinical presentation, including angina at rest associated with acute or transient ST segment or T wave changes in ECG without an increase in markers of myocardial injury (troponin T, myoglobin) and confirmation of significant coronary artery lumen stenosis (>70%) during coronary angiography. Patients with a final diagnosis of myocardial infarction based on a significant increase in markers of myocardial injury (troponin T, myoglobin) were excluded from the study. Patients with autoimmune diseases and cancer were also excluded from the study.

The control group consisted of 144 healthy subjects without a history of inflammatory disease or cancer. In this group of patients, no significant coronary lumen stenosis was detected at coronary angiography, performed for the diagnosis of unexplained chest pain. Clinical characteristics of patients and controls are shown in Table 1. The study was approved by the ethics committee at Pomeranian Medical University, Szczecin, Poland (KB-0012/46/17) and written informed consent was obtained from all subjects.

### 2.1. Genotyping

Genomic DNA was extracted from 1 mL of peripheral blood samples using a Genomic Mini AX Blood 1000 Spin kit (A&A Biotechnology, Gdynia, Poland) following manufacturers protocol. DNA was subsequently standardized to equal concentrations of 20 ng/μL, based on spectrophotometric absorbance measurement at 260/280 nm (DeNovix DS-11 FX+ Spectrophotometer/Fluorometer, Wilmington, DE, USA). Genotyping for the following single nucleotide polymorphisms (SNPs): *PECAM1* rs1867624, *COL4A2* rs4773144, *PHACTR1* rs9349379 and *LMOD1* rs2820315 was performed using a pre-validated allelic discrimination TaqMan real-time PCR assays (Life Technologies, Carlsbad, CA, USA) and TaqMan GTXpress Master Mix (Life Technologies, Carlsbad, CA, USA). Fluorescence data were captured using the ViiA7 Real-Time PCR System (Applied Biosystems, San Francisco, CA, USA) after 40 reaction cycles. Specific genotypes were assigned to individual samples after analysis with TaqMan Genotyper software (Thermo Fisher Scientific, Waltham, MA, USA).

### 2.2. Statistical Analysis

The concordance of genotype distributions with Hardy-Weinberg equilibrium (HWE) was assessed using Fisher’s exact test. The chi-square test was used to compare the distributions of genotypes and alleles between groups. The distribution of quantitative clinical parameters in the study group differed significantly from the normal distribution (Shapiro-Wilk test), so they were compared between groups using the non-parametric Mann-Whitney test. Multivariate logistic regression analysis, including the number of G alleles of the *PHACTR1* variant rs9349379 and the number of T alleles of the *LMOD1* variant rs2820315, as well as seven variables (age, sex, BMI, smoking, diabetes mellitus, hypertension, serum HDL cholesterol) as independent variables and the presence of unstable angina as the dependent variable, was performed to evaluate whether the *PHACTR1* rs9349379 and *LMOD1* rs2820315 polymorphisms were independent predictors of unstable angina. *p* < 0.05 was considered statistically significant.

The statistical power of study with 232 patients and 144 controls was sufficient to detect with 80% probability the real effect size of allele-phenotype association corresponding to odds ratio (OR) <0.64 or >1.53 for *COL4A2* rs4773144, *PECAM1* rs1867624, and *PHACTR1* rs9349379, and OR <0.59 or >1.59 for *LMOD1* rs2820315.

## 3. Results

The distributions of the studied polymorphisms are shown in Table 2 and Table 3. There were no statistically significant differences in the distribution of *COL4A2 rs4773144* and *PECAM1 rs1867624 gene polymorphisms between patients with unstable angina and control subjects.*

*In patients with unstable angina, there was an increased frequency of PHACTR1 rs9349379 G allele carriers (GG and AG genotypes)* (GG+AG vs. AA, OR 1.71; 95% CI 1.10–2.66, *p* = 0.017) and carriers of the *LMOD1* rs2820315 T allele (TT and CT genotypes) (TT+CT vs. CC, OR 1.65; 95% CI 1.09–2.51, *p* = 0.019) compared to the control group (Table 3).

To examine whether the *PHACTR1* rs9349379 *G allele and* *LMOD1* rs2820315 T allele are independent factors predisposing to unstable angina, multivariate regression analysis was performed including: age, male sex, BMI, tobacco smoking, hypertension, diabetes, HDL serum level, and the number of *PHACTR1* rs9349379 *G and* *LMOD1* rs2820315 T alleles. In this analysis, the higher number of *PHACTR1* rs9349379 *G and* *LMOD1* rs2820315 T alleles, as well as age, male sex, hypertension, higher BMI, diabetes and low HDL serum level, were independent factors predisposing to unstable angina (Table 4).

In addition, we examined the associations between the studied polymorphisms and clinical factors predisposing to unstable angina (age, BMI, waist circumference, serum levels of total cholesterol, HDL cholesterol, LDL cholesterol and triacylglycerols). These associations were not statistically significant (Kruskal-Wallis test) except for the association between *PHACTR1* rs9349379 genotype and HDL cholesterol level (Table 5). 

## 4. Discussion

The aim of this case-control association study was to evaluate the associations between the occurrence of acute coronary syndromes in the form of unstable angina and SNPs within the *PECAM1*, *COL4A2*, *PHACTR1* and *LMOD1* genes. These genes influence endothelial and vascular smooth muscle function and thus the development of the atherosclerotic process and the stability of the atherosclerotic plaque. The polymorphisms studied affect the expression of these genes and therefore may influence the atherosclerotic process and coronary artery disease. There were no significant differences in the distribution of *PECAM1* rs1867624 and *COL4A2* rs4773144 polymorphisms between patients with unstable angina and controls, suggesting that these polymorphisms do not increase the risk of unstable angina. In contrast, we found a statistically significant higher prevalence of the *PHACTR1* rs9349379 G allele and the *LMOD1* rs2820315 T allele in the group of patients with unstable angina. The association between these alleles and unstable angina was confirmed by multivariate logistic regression analysis, in which the number of the G (*PHACTR1* rs9349379) and T (*LMOD1* rs2820315) alleles was an independent risk factor for unstable angina. The results suggest an association between *PHACTR1* rs9349379 and *LMOD1* rs2820315 polymorphisms and the risk of unstable angina.

The gene encoding phosphatase and actin regulator 1, *PHACTR1*, is located on chromosomal locus 6p24. The rs9349379 polymorphism is located within an intron of the *PHACTR1* gene and is thought to be a regulatory region responsible for gene expression (eQTL—expression quantitative trait locus) in vascular tissue.

The present study demonstrates an association between the G allele and the risk of unstable angina. These results are consistent with previous studies that suggest an association of the G allele of rs9349379 with the development of ischaemic heart disease and the occurrence of myocardial infarction [7].

Beaudoin et al. showed that SNP rs9349379 is correlated with a defect in the binding site for the MEF2 (myocyte enhancer factor 2) family of transcription factors, which play a key role in maintaining the integrity of the vascular bed [8]. MEF2 is responsible for transmitting extracellular signals to the genome and activating pathways that control cell differentiation, morphogenesis, proliferation and apoptosis. It also mediates epigenetic regulatory mechanisms involving changes in chromatin configurations and microRNA modulation [9]. However, there are no consistent data to date showing a direct association between MEF2 expression and the development of CAD. In contrast, the different binding of the A and G alleles to MEF2 results in altered *PHACTR1* expression.

The G allele-associated defect in the binding site for MEF2 leads to decreased *PHACTR1* expression in the coronary artery endothelium [8]. The product of *PHACTR1* is protein phosphatase 1 (PP1) and actin binding protein. As a regulator of PP1 activity, it is highly expressed in the endothelium of the brain, heart and vasculature, playing an important role in tubulogenesis and endothelial apoptosis [10]. Among others, PP1 has a function in regulating endothelial NO production [11]. 

There are data showing an association between SNP rs9349379 and the development of coronary artery stenosis and calcification and early MI [12,13,14]. Pérez-Hernández et al. evaluated the effect of polymorphisms in the *PHACTR1* gene (rs2026458 and rs9349379) on the risk of early CAD development in a Mexican population. Analysis after adjustment for age, sex, presence of hypertension, type 2 diabetes, dyslipidaemia and nicotinism suggested an association between the rs9349379 G allele and an increased likelihood of CAD at a younger age [15]. Hager et al. evaluated the effect of SNP rs9349379 on the incidence of CAD in a Lebanese population. Their analysis showed an association between the rs9349379 G allele and the degree of coronary stenosis [14]. Consistent with these observations are the results of a study conducted in a Chinese population, where a reduced incidence of CAD was found among individuals with the A allele [16]. Gupta et al. observed an increase in endothelin-1 expression in the endothelium associated with the G allel [17]. This hormone is a potent endothelium-produced factor that causes vasoconstriction. Ford et al. also confirmed a positive correlation between the presence of the G allele and plasma endothelin-1 levels. They observed a higher prevalence of angina for this background associated with coronary microvascular dysfunction (CMD) in subjects without significant epicardial artery stenosis [18]. Kasikara et al. suggested that *PHACTR1* deficiency may exacerbate atherosclerotic progression. The authors demonstrated that decreased *PHACTR1* expression impairs the process of elimination of apoptotic remnants of dead macrophages called efferocytosis [19]. Efficient efferocytosis inhibits the inflammatory reaction and prevents secondary necrosis within the atherosclerotic plaque [19,20]. Reduced *PHACTR1* expression in macrophages reduces the phosphorylation of myosin light chains, which are essential for phagocytosis of apoptotic remnants. Consequently, there is a significant reduction in efferocytosis, which promotes the formation of rupture-prone necrotic atherosclerotic plaques [21].

*LMOD1* (leiomodin 1) belongs to the actin filament nucleator family and is found in smooth muscle cells, including VSMC. The gene encoding the protein is located at locus 1q32. LMOD2 and LMOD3 are not expressed in smooth muscle but are found in cardiac and skeletal muscle tissues [22]. Leiomodins are proteins that regulate actin filament function. They appear to play a significant role in maintaining the contractile function of striated and smooth muscle, and loss of these proteins significantly compromises the integrity of muscle tissue [23]. The current study found a significant increase in the risk of unstable angina in carriers of the *LMOD1* rs2820315 T allele. These observations are consistent with the results of GWAS analyses, which suggest that the rs2820315 polymorphism is significantly involved in the development of atherosclerotic lesions in coronary arteries [24]. The presence of the T allele is associated with reduced expression of *LMOD1* and leads to a defect in the transcription factor FOXO3 (forkhead box O3) binding site [25]. As a result, there is a decrease in its transcriptional activity and consequently a decrease in *LMOD1* concentrations. The rs2820315 T allele results in decreased expression of *LMOD1* in human coronary artery smooth muscle cells (HCASMC). Decreased expression leads to increased proliferation and migration and loss of contractile elements of VSMC. *LMOD1* activity is also decreased by oxLDL particles, PDGF-BB and some cytokines (INF-γ) [25,26].

Levula et al. evaluated gene expression in plaque fragments collected from femoral, carotid or abdominal aortic arteries. *LMOD1* expression was 6.5-fold lower compared with control samples from internal mammary arteries [27]. *LMOD1* appears to play a key role in maintaining the phenotype of differentiated VSMC and sustaining their contractile function. Its deficiency causes a loss of contractile elements and enhances VSMC differentiation into less mature cells with an embryonic stage-like phenotype. Epigenetic reprogramming of VSMC to a form exhibiting a high proliferation rate and invasiveness plays a key role in atherogenesis [28,29]. Studies on animal models convincingly demonstrate that up to more than 80% of all cells found in advanced atherosclerotic plaques are derived from VSMC. Phenotypically transformed muscle cells produce excess extracellular matrix, undergo apoptosis, accumulate lipids, secrete growth factors and proinflammatory cytokines, and contribute to monocyte recruitment [28].

*PECAM1* (*CD31*) encodes platelet-endothelial cell adhesion factor 1, a trans-membrane glycoprotein belonging to the immunoglobulin family found on the surface of platelets, monocytes and neutrophils. The *PECAM1* gene is located in the 17q23 region. PECAM-1 molecules interact with each other and bind to other molecules (cell-surface glycosaminoglycans, integrin αvβ3, CD38, TCD31L) [29,30].

Studies on the effect of polymorphisms in the *PECAM1* region on the risk of CAD and MI do not provide conclusive results [24]. The rs1867624 C allele is thought to be associated with higher *PECAM1* expression and lower CAD risk. *PECAM1* molecules are concentrated on the lateral surfaces of cell membranes. Mutual binding of *PECAM1* molecules to each other through so-called trans-homophilic junctions leads to the formation of a specific protective barrier of the endothelium. Downregulation of their expression leads to a violation of the integrity of the endothelial barrier and an increase in its permeability, which consequently leads to the activation of inflammatory processes in the inner membrane of the artery [31,32]. Previous studies suggested a dual function of *PECAM1* in the process of coronary arteriosclerosis. *PECAM1* enhances the signal transduction required for proinflammatory expression of adhesion molecules at sites prone to atherosclerotic lesion development and may inhibit inflammatory responses by inhibiting NF-κB expression, revealing its anti-atherosclerotic potential [33].

The *COL4A1*/*COL4A2* gene, which encodes type IV collagen, is located in the 13q34 region and includes exons 52 and 48. This type of collagen is a major building block of the basement membrane, anchoring, via integrins and other surface molecules, the epithelial cell layer of most tissues and the vascular endothelium. Collagen fibres, also type IV, are furthermore found in the endothelial membrane of arteries, surrounding densely packed VSMC. *COL4A1* encodes the α1 subunit. *COL4A2* encodes the α2 subunit, which is one of the six subunits of type IV collagen [34,35]. *COL4A1* and *COL4A2* genes are separated by a common bidirectional promoter. Expression of both genes is controlled by TGFβ, and a mediator necessary for this process is the SMAD3 protein. Studies indicate that transcription of *COL4A1* and *COL4A2* is modulated by non-coding regulatory intron sequences of these genes [34,35]. The SNP rs4773144 evaluated in this study is located within intron 3 of *COL4A2* [36]. GWAS studies suggest the involvement of rs4773144 *COL4A2* gene variants in the development of CAD and coronary artery calcification and increased risk of MI [12,37]. The results of the current study showed no association between the rs4773144 polymorphism and unstable angina.

In this study, we investigated genetic polymorphisms associated with an increased risk of coronary artery disease. To date, these polymorphisms were studied in various forms of coronary artery disease; however, the results are inconsistent. The results of our study suggest an association between *PHACTR1* rs9349379 and *LMOD1* rs2820315 polymorphisms and unstable angina. These genes affect endothelial and vascular smooth muscle function. It is likely that changes in the expression of these genes resulting from genetic polymorphisms may affect atherosclerotic plaque formation and stability and thus the risk of unstable angina. To date, several genetic polymorphisms that increase the risk of developing ischemic heart disease, including unstable angina, have been identified. It seems that genetic polymorphisms, together with well-known environmental risk factors, may be taken into account as factors influencing the occurrence of unstable angina.

## 5. Conclusions

The results of this study suggest that *PHACTR1* rs9349379 and *LMOD1* rs2820315 gene polymorphisms are associated with an increased risk of unstable angina. The presence of *PHACTR1* rs9349379 G and *LMOD1* rs2820315 T alleles is a risk factor for unstable angina. No statistically significant association was found between *PECAM1* rs1867624 and *COL4A2* rs4773144 gene polymorphisms and the risk of unstable angina.

## Figures and Tables

**Table 1:** Clinical characteristics of patients and control subjects.

Parameters	Control Group	Unstable Angina	*p* *
	Median (Q1–Q3)	Median (Q1–Q3)	
Age (ears)	67 (59–75)	61 (55–69)	<0.0001
BMI (kg/m^2^)	26 (24–28)	28 (26–31)	<0.0001
CH (mg/dL)	186 (167–213)	227 (187–267)	<0.0001
HDL (mg/dL)	54 (49–57)	43 (38–50)	<0.0001
LDL (mg/dL)	112 (87–138)	162 (127–190)	<0.0001
TG (mg/dL)	87 (74–132)	130 (86–172)	<0.0001
	N (%)	N (%)	*p* ^^^
Sex (male)	54 (37.5%)	172 (74.1%)	<0.0001
Arterial hypertension	57 (39.6%)	145 (62.5%)	<0.0001
Diabetes mellitus	9 (6.3%)	57 (24.6%)	<0.0001

* Mann-Whitney U test; ^^^ Fisher exact test; Q1—lower quartile, Q3—upper quartile; BMI—body mass index, CH—total cholesterol in serum, HDL—high density cholesterol in serum, LDL—low density cholesterol in serum, TG—triacylglycerols in serum.

**Table 2:** Distribution of *COL4A2* rs4773144 and *PECAM1* rs1867624 genotypes and alleles in patients with unstable angina and controls.

	Control Group		Unstable Angina		*p* Value ^^^	Compared Genotypes or Alleles	*p* Value ^^^	OR (95% CI)
	n	%	n	%				
*COL4A2 rs4773144* genotype								
AA	49	34.03%	63	27.16%	0.35	GG+AG vs. AA	0.16	1.38 (0.88–2.17)
AG	72	50.00%	125	53.88%		GG vs. AG+AA	0.46	1.23 (0.71–2.14)
GG	23	15.97%	44	18.97%		GG vs. AA	0.21	1.49 (0.79–2.79)
						AG vs. AA	0.21	1.35 (0.84–2.17)
						GG vs. AG	0.74	1.10 (0.62–1.97)
Allele								
A	170	59.03%	251	54.09%		G vs. A	0.19	1.22 (0.91–1.65)
G	118	40.97%	213	45.91%				
*PECAM1 rs1867624* genotype								
TT	44	30.56%	74	31.90%	0.19	CC+TC vs. TT	0.79	0.94 (0.60–1.47)
TC	60	41.67%	112	48.28%		CC vs. TC+TT	0.074	0.64 (0.40–1.05)
CC	40	27.78%	46	19.83%		CC vs. TT	0.19	0.68 (0.39–1.20)
						TC vs. TT	0.67	1.11 (0.68–1.81)
						CC vs. TC	0.071	0.62 (0.36–1.04)
Allele								
T	148	51.39%	260	56.03%		C vs. T	0.21	0.83 (0.62–1.11)
C	140	48.61%	204	43.97%				

^^^ χ^2^ test; HWE: control group *p* = 0.73, unstable angina *p* = 0.23 for *COL4A2 rs4773144*; HWE: control group *p* = 0.05, unstable angina *p* = 0.79 for *PECAM1 rs1867624*.

**Table 3:** Distribution of *PHACTR1* rs9349379 and *LMOD1 rs2820315* genotypes and alleles in patients with unstable angina and controls.

	Control Group		Unstable Angina		*p* Value ^^^	Compared Genotypes or Alleles	*p* Value^^^	OR (95% CI)
	n	%	n	%				
*PHACTR1* rs9349379 genotype								
AA	56	38.89%	63	27.16%	0.041	GG+AG vs. AA	0.017	1.71 (1.10–2.66)
AG	70	48.61%	126	54.31%		GG vs. AG+AA	0.12	1.59 (0.88–2.89)
GG	18	12.50%	43	18.53%		GG vs. AA	0.024	2.12 (1.10–4.10)
						AG vs. AA	0.046	1.60 (1.01–2.54)
						GG vs. AG	0.37	1.33 (0.71–2.47)
Allele								
A	182	63.19%	252	54.31%		G vs. A	0.017	1.44 (1.07–1.95)
G	106	36.81%	212	45.69%				
*LMOD1* rs2820315 genotype								
CC	80	55.56%	100	43.10%	0.018	TT+CT vs. CC	0.019	1.65 (1.09–2.51)
CT	57	39.58%	105	45.26%		TT vs. CT+CC	0.026	2.58 (1.09–6.09)
TT	7	4.86%	27	11.64%		TT vs. CC	0.0094	3.09 (1.28–7.45)
						CT vs. CC	0.081	1.47 (0.95–2.28)
						TT vs. CT	0.10	2.09 (0.86–5.11)
Allele								
C	217	75.35%	305	65.73%		T vs. C	0.0054	1.59 (1.15–2.22)
T	71	24.65%	159	34.27%				

^^^ χ^2^ test; HWE: control group *p* = 0.72, unstable angina *p* = 0.19 for *PHACTR1* rs9349379; HWE: control group *p* = 0.51, unstable angina *p* = 1.00 for *LMOD1* rs2820315.

**Table 4:** Multivariate logistic regression analysis using unstable angina as the dependent variable.

Parameter	OR (95% CI)	*p* Value
Age (years)	0.96 (0.93–0.99)	0.022
Sex (male vs. female)	4.60 (2.25–9.39)	0.000026
BMI (kg/m^2^)	1.20 (1.09–1.32)	0.00014
Smoking	2.06 (0.94–4.49)	0.069
Arterial hypertension	2.16 (1.07–4.39)	0.032
Diabetes mellitus	5.47 (1.89–15.82)	0.0016
HDL (mg/dL)	0.88 (0.84–0.92)	<0.00001
*PHACTR1* rs9349379 (number of G alleles)	1.70 (1.04–2.79)	0.034
*LMOD1* rs2820315 (number of T alleles)	1.99 (1.18–3.37)	0.0098

**Table 5:** Clinical features of patients with unstable angina stratified according to *PHACTR1* rs9349379 genotypes.

Parameters	*PHACTR1* rs9349379 Genotype													
		AA		AG		GG		AA+AG		AG+GG	*p* ^#^	AA vs. AG+GG	AA+AG vs. GG	AA vs. GG
	n	Mean ± SD	n	Mean ± SD	n	Mean ± SD	n	Mean ± SD	n	Mean ± SD		*p* ^&^		
Age (years)	63	62.6 ± 9.9	126	61.4 ± 8.9	43	63.1 ± 11.4	189	61.8 ± 9.2	169	61.9 ± 9.6	0.54	0.62	0.42	0.74
BMI (kg/m^2^)	63	27.9 ± 3.7	126	28.4 ± 4.0	43	28.9 ± 4.1	189	28.3 ± 3.9	169	28.5 ± 4.0	0.58	0.41	0.40	0.29
Waist (cm)	63	93.0 ± 10.0	126	96.0 ± 10.7	43	95.3 ± 10.0	189	95.0 ± 10.5	169	95.8 ± 10.5	0.27	0.15	0.79	0.54
CH (mg/dL)	61	234.2 ± 54.0	120	225.9 ± 55.1	42	237.0 ± 62.5	181	228.7 ± 54.7	162	228.8 ± 57.1	0.33	0.32	0.45	0.95
HDL (mg/dL)	50	44.3 ± 9.5	103	43.9 ± 8.1	33	48.4 ± 6.8	153	44.0 ± 8.5	136	45.0 ± 8.0	0.01	0.34	0.002	0.01
LDL (mg/dL)	50	168.9 ± 47.9	103	159.7 ± 50.8	33	168.4 ± 53.8	153	162.7 ± 49.9	136	161.8 ± 51.5	0.34	0.24	0.61	0.74
TG (mg/dL)	61	139.7 ± 80.6	119	138.2 ± 74.2	42	144.3 ± 59.7	180	138.7 ± 76.2	161	139.8 ± 70.6	0.58	0.63	0.30	0.31

^#^—Kruskal-Wallis test; ^&^—Mann-Whitney U test; BMI—body mass index, CH—total cholesterol in serum, HDL—high density cholesterol in serum; LDL—low density cholesterol in serum, TG—triacylglycerols in serum.

## Data Availability

Not applicable.
